# Pathogenic Bacteria Target NEDD8-Conjugated Cullins to Hijack Host-Cell Signaling Pathways

**DOI:** 10.1371/journal.ppat.1001128

**Published:** 2010-09-30

**Authors:** Grégory Jubelin, Frédéric Taieb, David M. Duda, Yun Hsu, Ascel Samba-Louaka, Rika Nobe, Marie Penary, Claude Watrin, Jean-Philippe Nougayrède, Brenda A. Schulman, C. Erec Stebbins, Eric Oswald

**Affiliations:** 1 INRA, UMR 1225, Toulouse, France; 2 Université de Toulouse; ENVT; UMR 1225; Toulouse, France; 3 Howard Hughes Medical Institute, St. Jude Children's Research Hospital, Department of Structural Biology, Memphis, Tennessee, United States of America; 4 Laboratory of Structural Microbiology, Rockefeller University, New York, New York, United States of America; 5 Université de Toulouse; UPS; Faculté de Médecine; Toulouse, France; 6 CHU de Toulouse; Institut Fédératif de Biologie; Laboratoire de Bactériologie-Hygiène; Toulouse, France; Institut Pasteur, France

## Abstract

The cycle inhibiting factors (Cif), produced by pathogenic bacteria isolated from vertebrates and invertebrates, belong to a family of molecules called cyclomodulins that interfere with the eukaryotic cell cycle. Cif blocks the cell cycle at both the G_1_/S and G_2_/M transitions by inducing the stabilization of cyclin-dependent kinase inhibitors p21^waf1^ and p27^kip1^. Using yeast two-hybrid screens, we identified the ubiquitin-like protein NEDD8 as a target of Cif. Cif co-compartmentalized with NEDD8 in the host cell nucleus and induced accumulation of NEDD8-conjugated cullins. This accumulation occurred early after cell infection and correlated with that of p21 and p27. Co-immunoprecipitation revealed that Cif interacted with cullin-RING ubiquitin ligase complexes (CRLs) through binding with the neddylated forms of cullins 1, 2, 3, 4A and 4B subunits of CRL. Using an *in vitro* ubiquitylation assay, we demonstrate that Cif directly inhibits the neddylated CUL1-associated ubiquitin ligase activity. Consistent with this inhibition and the interaction of Cif with several neddylated cullins, we further observed that Cif modulates the cellular half-lives of various CRL targets, which might contribute to the pathogenic potential of diverse bacteria.

## Author Summary

Among the arsenal of virulence factors used by bacterial pathogens to infect and manipulate their hosts, cyclomodulins are a growing family of bacterial toxins that interfere with the eukaryotic cell-cycle. Cif is one of these cyclomodulins produced by both mammalian and invertebrate pathogenic bacteria. Cif blocks the host cell cycle by inducing the accumulation of two regulators of cell cycle progression: the cyclin-dependent kinase inhibitors p21 and p27. To decipher the mode of action of Cif, we performed yeast two-hybrid screenings. We show that Cif binds to NEDD8 and induce accumulation of neddylated cullins early after infection. Cullins are scaffold components of cullin-RING ubiquitin ligases (CRLs), which ubiquitinate proteins and target them for degradation by the 26S proteasome. We demonstrate that Cif directly inhibits the ubiquitin ligase activity of these CRLs and consequently the targeting of p21 and p27 for ubiquitin-dependent degradation. Targeting at NEDD8 represents a novel strategy for modulation of host cell functions by bacterial pathogens. By inhibiting the most prominent class of ubiquitin-ligases, Cif controls the stability of a cohort of key regulators and impinge on not only cell cycle progression but also on many cellular and biological processes such as immunity, development, transcription, and cell signaling.

## Introduction

Cycle inhibiting factors (Cif) constitute a family of bacterial cyclomodulins that inhibit eukaryotic cell proliferation by blocking the cell cycle [1], [2], [3]. First identified in enteropathogenic and enterohemorrhagic *Escherichia coli* (EPEC and EHEC) [4], Cif homologues have been recently characterized in several other pathogenic proteobacteria including *Burkholderia*, *Yersinia* and *Photorhabdus* species [5], [6]. *E. coli* Cif is composed of a C-terminal active domain and a ∼20 amino acid N-terminal signal domain necessary for its injection into host cells by the type three secretion system (T3SS) [7], [8]. Crystal structures of Cif from *E. coli*, *B. pseudomallei* and *P. luminescens* revealed that Cif proteins possess a conserved putative catalytic triad composed of a cysteine, a histidine and a glutamine, and belong to the cysteine protease superfamily, a diverse group of enzymes with protease, acetyltransferase, deamidase, transglutaminase, and other biochemical activities [5], [9], [10]. Although the specific enzymatic activity of Cif has not been identified, the active site is essential for Cif cytostatic activity since mutation of the triad residues leads to the loss of phenotype [5], [6], [10].

Upon injection into host cells, Cif triggers a cytopathic effect characterized by cell cycle arrests both in G_1_/S and G_2_/M phase transitions and, in certain cell lines, the reorganization of the actin network [4], [11], [12]. The cell cycle arrest induced by Cif is irreversible and leads eventually to delayed cell death by apoptosis [13]. The inhibition of the cell cycle is independent of the DNA damage response and p53 pathway [14], but correlates with the accumulation of the cyclin-dependent kinase (CDK) inhibitors p21^waf1/cip1^ and p27^kip1^ (hereafter referred as p21 and p27 respectively) that inactivate CDKs whose activities are required for entry in both S- and M-phases [15], [16]. This accumulation of p21 and p27 results from protein stabilization, suggesting that Cif interferes with their 26S proteasome-mediated degradation [15].

In eukaryotic cells, degradation of intracellular proteins is processed mainly by ubiquitin-mediated proteolysis [17]. This highly regulated mechanism leads to conjugation of polyubiquitin chains to protein substrates, targeting them to the 26S proteasome for degradation [18]. Substrate specificity is determined by E3 ligase enzymes that recognize targets to be ubiquitinated. The most prominent class of E3s is the superfamily of cullin-RING ligases (CRLs) that are multi-subunit complexes based on cullin protein scaffolds. Among the different mechanisms regulating the activity of CRLs, conjugation of the cullin subunit with the ubiquitin-like protein NEDD8 (neddylation) stimulates the transfer of ubiquitin to target proteins, thereby enhancing their subsequent proteolysis by the 26S proteasome [19], [20], [21]. Degradation of many proteins involved in the control of cell cycle progression, including p21 and p27, is regulated by CRL activity [22].

To decipher the mode of action of the cyclomodulin Cif, we performed yeast two-hybrid screenings and identified NEDD8 as a specific Cif interaction partner. Cif co-compartmentalized with NEDD8 in the host cell nucleus and induced an accumulation of NEDD8-conjugated cullins that correlated with the accumulation of p21 and p27. Co-immunoprecipitation revealed that Cif interacted with the neddylated forms of cullin-RING complexes. Morevover, *in vitro*, Cif inhibited NEDD8-modified Cullin1/Fbw7-associated ubiquitin ligase activity. Thus, Cif manipulates the host ubiquitin-dependent proteasomal degradation by interfering with NEDD8-conjugated CRLs.

## Results

### Cif interacts with the ubiquitin-like protein NEDD8

To identify eukaryotic targets of Cif, yeast two-hybrid screenings were performed using the wild-type form of *E. coli* Cif protein, an inactive cysteine mutant [10] and two distinct cDNA libraries from human placenta and human colon. The neural precursor cell expressed, developmentally down-regulated 8 protein (NEDD8) was the sole common putative eukaryotic interacting partner detected in each screening. In the two-hybrid, the highest confidence score, which reflects the reliability and biological significance of each interaction [23], was obtained for NEDD8 interacting with the cysteine mutant, suggesting that inactivation of the Cif catalytic site could stabilize the interaction with NEDD8.

**Figure 1:**
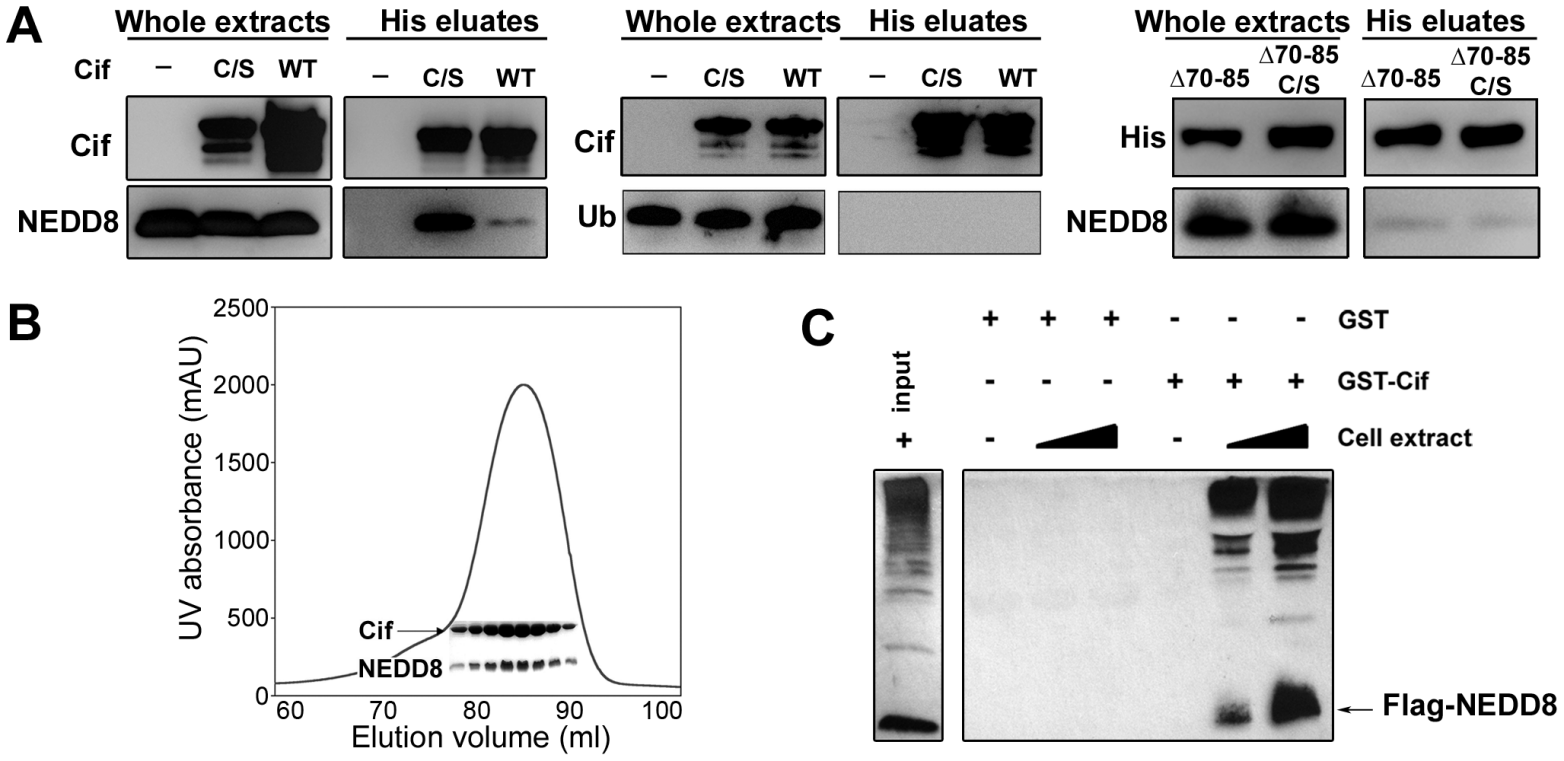
Cif interacts *in vitro* with the ubiquitin-like protein NEDD8. (**A**) Hexa-histidine tagged wild-type Cif (WT), Cif cysteine 109 mutant Cys-to-Ser (C/S), Cif deleted for amino-acids 70-85 mutant (Δ70-85) or Cif Δ70-85 Cys-to-Ser mutant(Δ70-65 C/S) and NEDD8 or ubiquitin (Ub) proteins were co-expressed in bacteria, and then His-Cif proteins were purified from bacterial lysates using nickel affinity. Whole extracts and eluted fractions were probed with indicated antibodies. (**B**) Wild-type His-Cif and NEDD8 were co-expressed in bacteria and His-Cif was purified using nickel affinity and gel filtration chromatography. The peak in the chromatograph corresponds to the His-Cif complex. NEDD8 and the corresponding fractions were visualized using SDS-PAGE stained with Coomassie blue. (**C**) Glutathione sepharose matrix was mixed with 100 µg of GST or GST-Cif Cys-to-Ser mutant purified proteins and then incubated with a lysate of HEK293T cells expressing a FLAG-NEDD8 construct. 100 µg or 250 µg of cells extract were used to obtain a GST constructs/cell extract ratio of 1∶1 and 1∶2.5 respectively. Cells extract (input) and eluted fractions were immunoblotted with anti-FLAG antibodies. FLAG-NEDD8 fusion protein is indicated with an arrow.

As a first step to validate the interaction between Cif and NEDD8, hexa-histidine tagged Cif (wild type or cysteine mutant) and NEDD8 were co-expressed in a laboratory *E. coli* strain and His-Cif was purified by nickel affinity chromatography. As a control, NEDD8 was also expressed in absence of His-Cif. Detection of NEDD8 in the eluted fractions from both wild type Cif and cysteine mutant purifications confirmed that Cif interacted with NEDD8 (Fig. 1A). Mirroring the yeast two-hybrid experiments, the cysteine mutant was more efficient to pull-down NEDD8 in the bacterial co-expression assay. In addition, Cif and NEDD8 co-eluted in the same fractions by gel filtration chromatography (Fig. 1B). Since NEDD8 is 60% identical and 80% homologous to ubiquitin, we also tested whether Cif could bind ubiquitin. In contrast to NEDD8, ubiquitin did not co-elute with His-Cif in the bacterial co-expression assay (Fig. 1A), demonstrating the specificity of Cif binding to NEDD8.

As a second approach to confirm the interaction of Cif with NEDD8, lysates from HEK293T cells expressing FLAG-tagged NEDD8 were used to perform glutathione S-transferase (GST) pull-down experiments. The glutathione-sepharose matrix was first loaded with purified GST-Cif (cysteine mutant) fusion protein or GST alone as control, and then incubated with cell extracts. In contrast to the GST control, GST-Cif captured FLAG-NEDD8 and apparently FLAG-NEDD8-modified proteins present in the HEK293T extract (Fig. 1C). Together, these results demonstrate that Cif interacts specifically with the NEDD8 protein.

**Figure 2:**
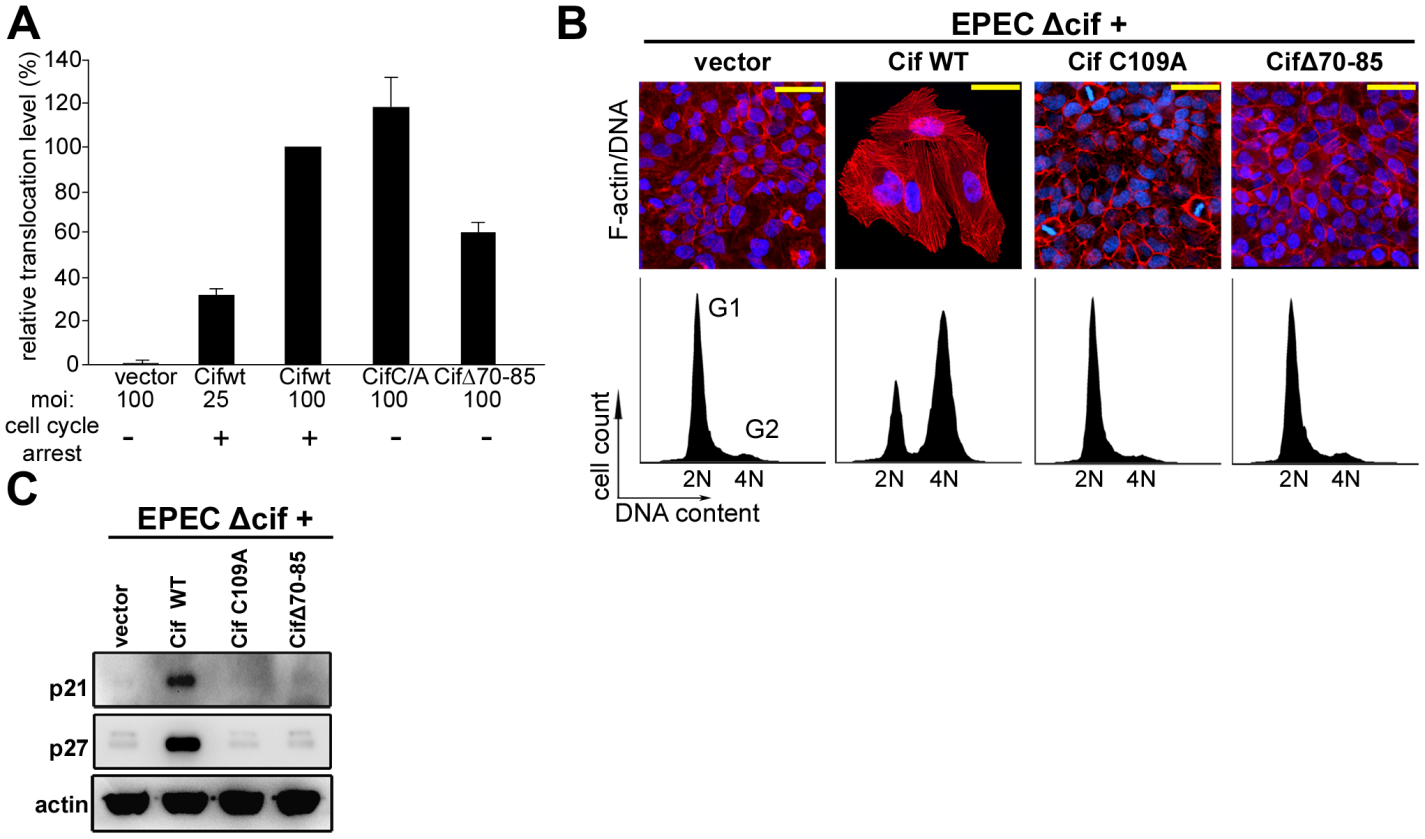
Cytopathic activities of wild-type Cif, Cif C/A and CifΔ70-85 mutants. (**A**) Translocation efficiency of Cif wild-type and mutant proteins were assessed as follow: HeLa cells were loaded with CCF2/AM substrate and were infected 1 h 30 with E22Δ*cif* hosting plasmids expressing the different Cif-TEM1 fusions and intracellular β-lactamase activity was detected by measuring cleavage of the CCF2/AM substrate. Translocation level is represented as percentages relative to translocation of the Cif WT-TEM protein (see materials and methods). Experiments were performed in triplicate and error bars represent standard errors of the mean. The multiplicity of infection (moi) and corresponding cell cycle arrest phenotype are indicated. Note that infection at moi of 25 with low level of translocation of wild-type Cif was sufficient to induce cell cycle arrest. (**B**) G_1_/S synchronized HeLa cells were infected 1.5 h with E22Δ*cif* expressing the different Cif proteins, washed and incubated with antibiotic for 20-72 h. Upper panels: F-actin was labeled with phalloidin-rhodamine (red) and DNA with DAPI (blue) 72 h post-infection. Bars represent 50 µm. Lower panels: cell cycle distribution was analyzed by flow cytometry 20 h post-infection. 2N (G1) and 4N (G2) populations are indicated. (**C**) HeLa cells were infected as in B, washed and incubated with antibiotics for 24 h. Cell protein extracts were probed with anti-p21, anti-p27 and anti-actin antibodies.

While members of the Cif family exhibit a low level of sequence similarity, crystal structure determination of Cif from *E. coli* and the xenologs from *B. pseudomallei* and *P. luminescens* revealed that all proteins are structurally well-conserved [5], [9], [10]. These studies have shown a head-and-tail domain arrangement. The tail corresponds to the N-terminal part of the protein. The C-terminal head-domain, which contains the catalytic triad, adopts an overall fold similar to cysteine proteases. Interestingly, deletion of the protruding α4 helix in the N-terminal part of Cif from *B. pseudomalei*, which is unlikely to affect the structural integrity of protein [5], abolishes the cell cycle arrest, suggesting that this domain could mediate substrate recognition [5]. Therefore, we constructed an *E. coli* Cif mutant deleted for residues 70 to 85 corresponding to the α4 helix (CifΔ70-85). The CifΔ70-85 mutant, like the Cif cysteine mutant, was translocated into host cells upon infection (Fig. 2A). However, removal of the α4 helix of Cif, even in combination with the cysteine mutation that increases *in vitro* NEDD8 binding efficiency, prevented association with NEDD8 (Fig. 1A). Furthermore, the CifΔ70-85 mutant did not induce actin stress fibers, nucleus enlargement, cell cycle arrest, or p21 and p27 accumulation (Fig 2B–C). Together these results suggest that interaction with NEDD8 via residues 70–85 is required for Cif-induced cell cycle arrest.

### Cif co-compartmentalizes with NEDD8 in the host cell nucleus

**Figure 3:**
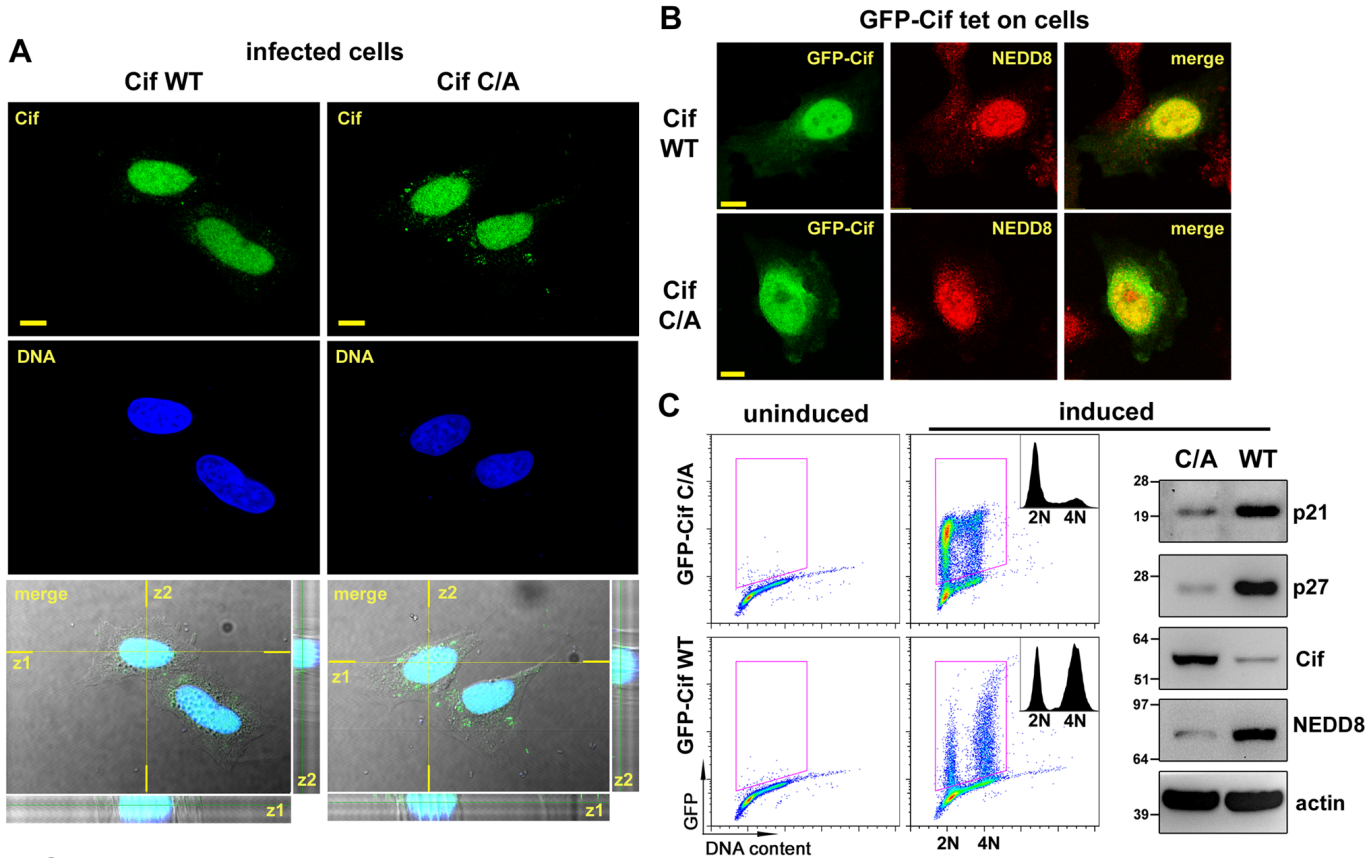
Cif is sorted to the host cell nucleus and co-compartmentalizes with NEDD8. (**A**) HeLa cells were infected 2 h with EPEC*Δcif* expressing wild-type Cif (WT) or the CifC109A (C/A) mutant, washed and further incubated 3 h with antibiotics. Cif was then visualized by indirect immunofluorescence and confocal imaging, with anti-Cif antibodies (green) and TO-PRO-3 to stain DNA (dark blue). Single optical XY slices and the corresponding Z slices of the image stacks are shown. In the merged images, coincident fluorescent sources appear cyan. Bars represent 10 µm. (**B**) Stable HeLa TetOn cells expressing GFP-Cif (WT) or GFP-CifC109A (C/A) were induced for 24 h with doxycycline. GFP-Cif was visualized by GFP fluorescence acquisition (green) and NEDD8 was detected by indirect immunofluorescence with anti-NEDD8 antibodies (red). In the merged images, coincident fluorescent sources appear orange-yellow. Bars represent 10 µm. (**C**) HeLa TetOn cells were induced or not with doxycycline, then DNA was stained with propidium iodide and DNA/GFP content was analyzed by flow cytometry. GFP-positive cells were gated as shown in the DNA content/GFP fluorescence 2D plots to generate the cell cycle histograms (insets). 2N and 4N DNA content are indicated. Induced cell protein lysates were probed with anti-p21, anti-p27, anti-Cif, anti-NEDD8 and anti-actin antibodies. Molecular weights in kDa are indicated.

Determination of Cif trafficking upon bacterial injection into host cells may provide clues to specify cellular functions hijacked by Cif. We therefore examined the subcellular localization of Cif into infected host cells by immunostaining and confocal microscopy. Optical sections revealed that Cif accumulated mostly in the host cell nucleus (Fig. 3A). A similar distribution of the inactive Cif cysteine mutant was observed. As a second step, we investigated whether Cif and NEDD8 co-localized in cells. Experiments were performed with stable HeLa Tet-on cells producing GFP-Cif (wild-type or cysteine mutant) fusion proteins. The distribution of the GFP signal showed that, as during infection experiments, wild-type Cif and the inactive cysteine mutant were mostly detected in the nucleus (Fig. 3B). In agreement with previous studies [24], immunodetection of NEDD8 showed its predominant localization also in the nucleus. Consistent with the nuclear localization of both proteins, dual GFP-Cif and NEDD8 labeling resulted in a partial coincident nuclear staining pattern (Fig. 3B). As expected, expression of GFP-Cif wild-type led to cell cycle arrest and to p21 and p27 accumulation whereas GFP-Cif cysteine mutant did not (Fig. 3C). These data demonstrate that Cif preferentially localizes to host cell nuclei, thus co-compartmentalizing with NEDD8.

### Cif induces accumulation of neddylated proteins in host cells

**Figure 4:**
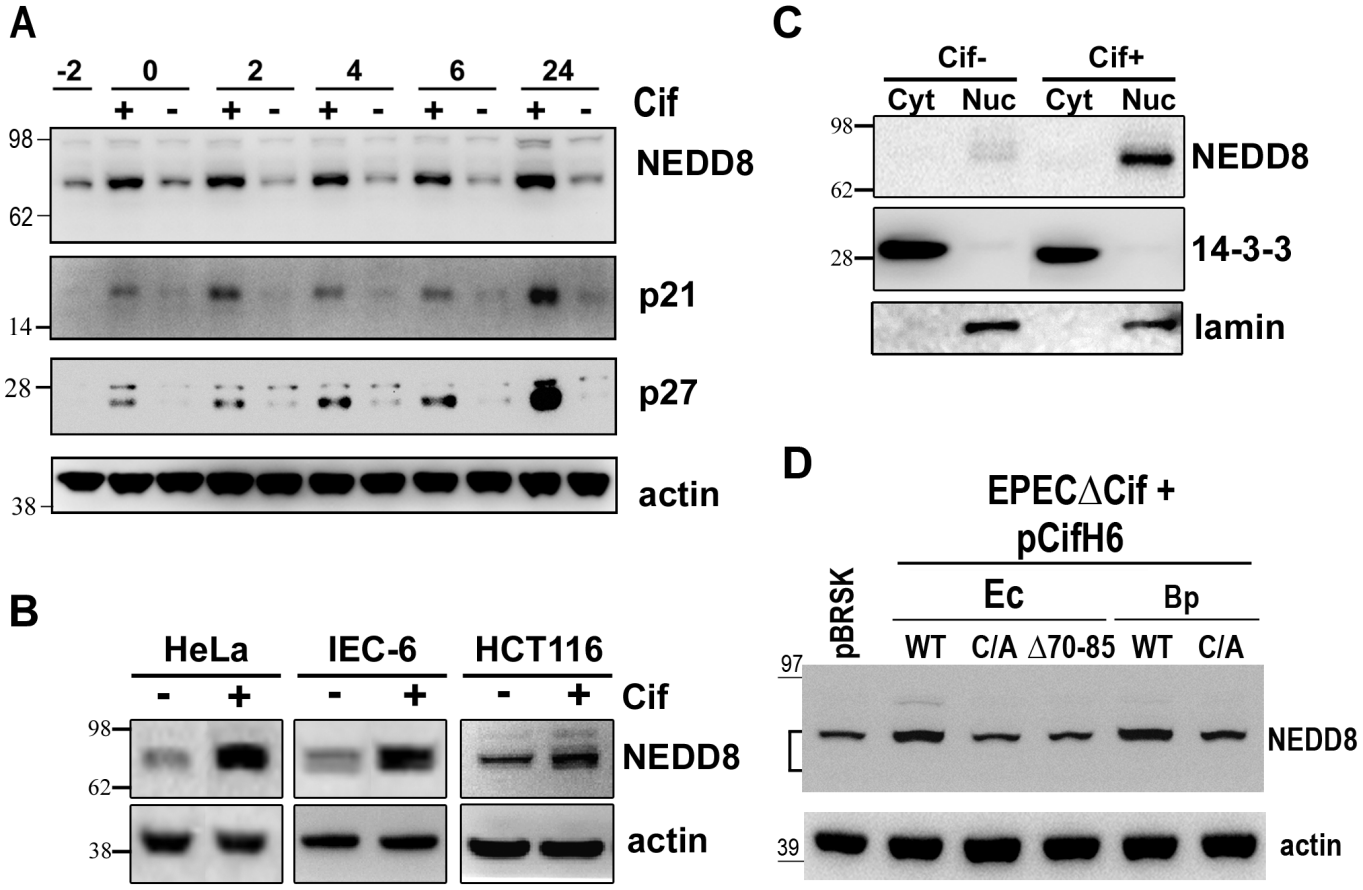
Infection with Cif-producing EPEC induces the accumulation of neddylated proteins. (**A**) HeLa cells were infected (starting at -2 h) 2 h with wild-type EPEC (+) or a Cif-deleted mutant EPEC (-), washed and further incubated with antibiotics up to 24 h. Cell protein extracts were probed with anti-NEDD8, anti-p21, anti-p27 and anti-actin antibodies. Molecular weights in kDa are indicated. (**B**) HeLa, IEC-6 or HCT116 cells were infected as in A and further incubated 6 h with antibiotics. Cell protein extracts were probed with anti-NEDD8 and anti-actin antibodies. (**C**) HeLa cells were infected as in A, washed and further incubated 6 h with antibiotics. Cytoplasmic (Cyt) and nuclear (Nuc) extracts were probed with anti-NEDD8, anti-14-3-3 (cytoplasmic control) and anti-lamin (nuclear control) antibodies. (**D**) HeLa cells were infected 3 h with EPECΔ*cif* strain carrying the empty vector (pBRSK) or plasmids coding for His-tagged wild-type (WT), cysteine mutant (C/A) or Δ70-85 mutant of Cif from *E. coli* (Ec) or from *B. pseudomallei* (Bp) and further incubated 3 h with antibiotics. Cell protein extracts were probed with anti-NEDD8 and anti-actin antibodies. The brackets encompass the 73-81 kDa region.

To investigate the biological significance of the Cif/NEDD8 interaction, we monitored the level of neddylated proteins in cells infected with wild type EPEC or strains deleted for the Cif gene (Δ*cif*). Immunoblotting with anti-NEDD8 antibodies revealed that the level of neddylated protein(s) with an apparent size of ∼80 kDa increased in cells infected with wild-type EPEC, but not with the Δ*cif* mutant strain (Fig. 4A). Accumulation of these neddylated proteins was detectable as soon as the end of infection (0 h) and was still observable 24 h post-infection. The increased level of neddylated proteins correlated with accumulation of p21 and p27 (Fig. 4A). The rapid accumulation of NEDD8-conjugated proteins was also observed in non-transformed rat small intestine epithelial IEC-6 cells and in human colon HCT116 cells (Fig. 4B). This accumulation correlated with their cell cycle arrest upon infection with EPEC expressing Cif [15]. Consistent with Cif and NEDD8 cellular localization (Fig. 3B), cell fractionation revealed that the 80 kDa neddylated proteins were mostly detected in the nuclear compartment (Fig. 4C). Cif alone was sufficient to modulate the level of neddylated proteins since eukaryotic expression of the wild-type GFP-Cif in the Tet-on cells also led to accumulation of the 80 kDa NEDD8-conjugated proteins (Fig. 3C). The Cif from *B. pseudomallei*, the most divergent xenolog of Cif, also induced accumulation of the 80 kDa neddylated proteins (Fig. 4D). In contrast, the level of NEDD8-conjugated proteins did not increase in cells infected with bacteria expressing Cif mutated on the catalytic cysteine, or the Cif mutant deleted for the α4helix (CifΔ70-85). Thus, a functional Cif was required for neddylated protein accumulation (Fig. 4D). Together, these data demonstrate that Cif alters the neddylation pattern in host cells and provokes an early accumulation of NEDD8-conjugated protein(s) in the nucleus of infected cells.

### Neddylated cullins 1, 2, 3, 4A and 4B accumulate in the presence of Cif

**Figure 5:**
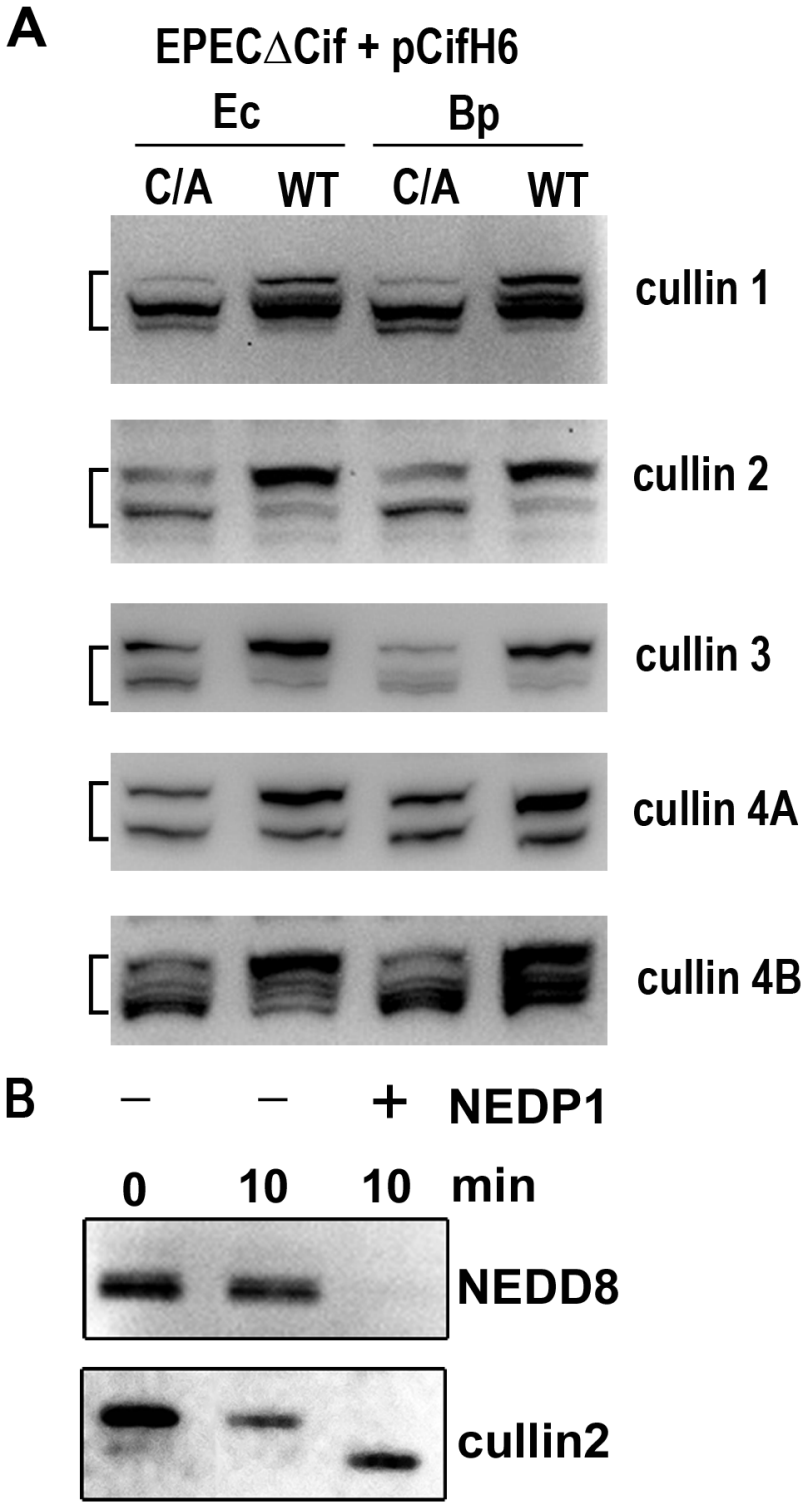
Cif induces the accumulation of NEDD8-conjugated cullins in host cells. (**A**) HeLa cells were infected 3 h with EPECΔ*cif* strain carrying plasmids coding for His-tagged wild-type (WT) or cysteine mutant (C/A) from *E. coli* (Ec) or from *B. pseudomallei* (Bp) and further incubated 3 h with antibiotics. Cell protein extracts were probed with anti-cullins (cullin 1 to cullin 4B) antibodies. The brackets encompass the 73-81 kDa regions. (**B**) Protein extracts from HeLa cells infected with EPEC expressing wild-type Cif were incubated for 10 min in presence (+) or absence (-) of NEDP1 and probed with anti-NEDD8 and anti-cullin2 antibodies.

Among known NEDD8-conjugated proteins, members of the cullin protein family are major substrates of the neddylation machinery [25]. Most eukaryotic genomes encode at least six cullins (CUL1, 2, 3, 4A, 4B and 5) that are scaffold proteins of cullin-RING E3 ubiquitin ligases. Since most of the cullin proteins are known to compartmentalize in the nucleus and have an approximate molecular weight of 80 kDa, we examined whether the Cif-induced neddylated proteins corresponded to several of the cullins (CUL1, 2, 3, 4A and 4B) [26]. The neddylation status of these cullins was investigated by immunoblotting following infection with EPEC Δ*cif* exogenously expressing Cif proteins (wild-type or cysteine mutants) from *E. coli* or *B. pseudomallei*. As expected, two bands were detected for the cullin proteins, with estimated molecular weights of ∼75 and a slower migrating form of ∼80 kDa (Fig. 5A). The slower migrating species corresponded to the signal detected with the anti-NEDD8 antibody, and treatment of cell extracts with the NEDD8 protease 1 (NEDP1) induced disappearance of both the higher molecular form of cullins and the corresponding NEDD8 signal (Fig. 5B), indicating that these slower migrating proteins were neddylated cullins. Following infection, the levels of neddylated forms of CUL1, 2, 3, 4A and 4B increased in presence of the wild-type Cif proteins from *E. coli* or from *B. pseudomallei* (Fig. 5A). These findings indicated that the 80 kDa neddylated proteins accumulating in presence of Cif were proteins of the cullin family.

### Cif interacts with neddylated cullin-RING complexes

**Figure 6:**
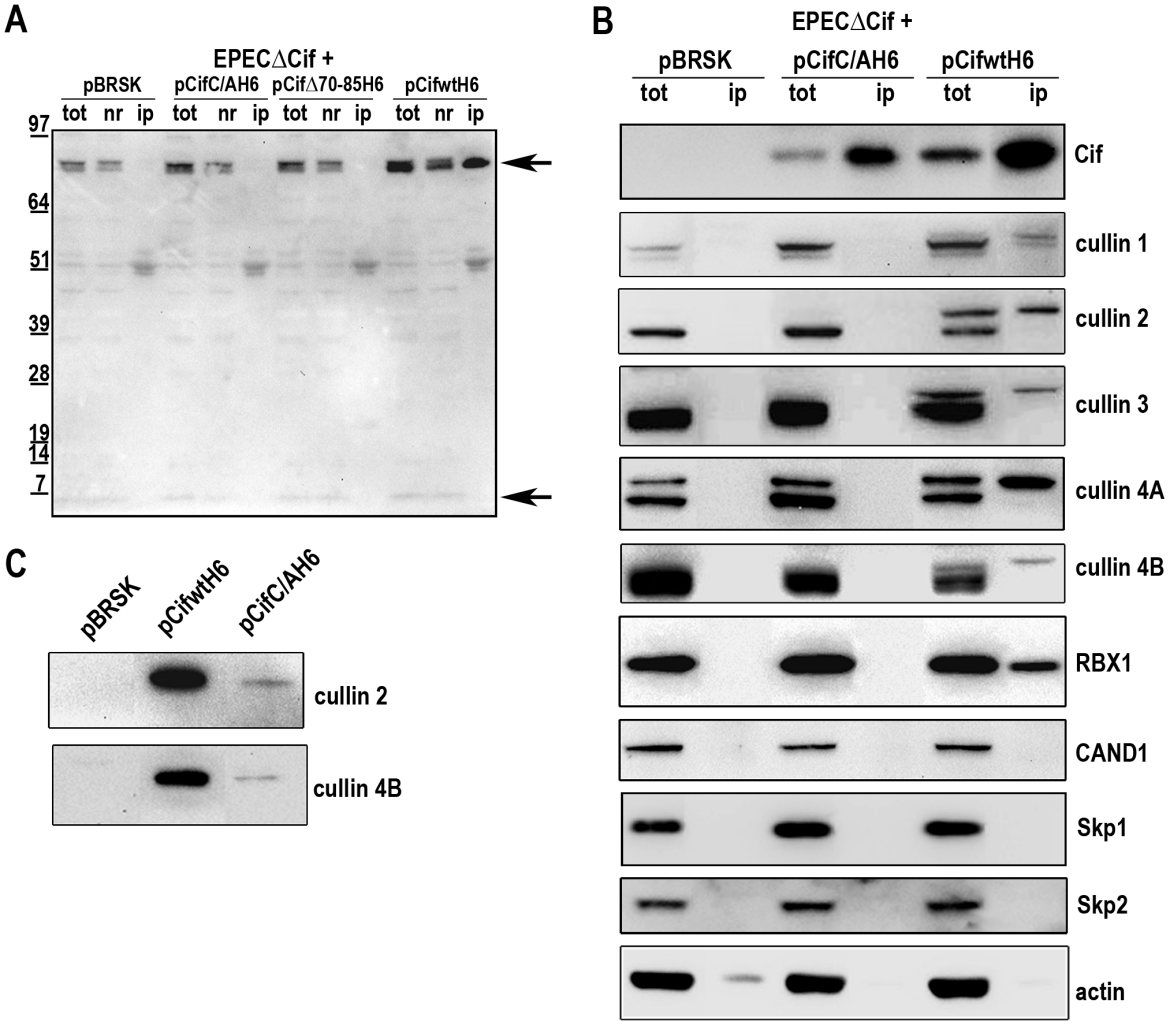
Cif interacts with NEDD8-conjugated cullins. HeLa cells were infected 3 h with EPEC*Δcif* carrying the empty vector (pBRSK) or plasmids expressing His-tagged *E. coli* Cif cysteine mutant (C/A), 70-85 deleted mutant (Δ70-85) or wild-type (WT) and further incubated 3 h with antibiotics. Cell extracts were immunoprecipitated with anti-His antibodies. (**A**) Total (tot), non-retained (nr) and immunoprecipitated (ip) protein fractions were probed with anti-NEDD8 antibodies. Arrows indicate neddylated cullins (top) and monomeric NEDD8 (bottom). Molecular weights in kDa are indicated. (**B**) Total and immunoprecipitated protein fractions were probed with anti-Cif, anti-cullins (CUL1 to CUL4B), anti-RBX1, anti-CAND1, anti-Skp1, anti-Skp2 and anti-actin antibodies. (**C**) High loads of immunoprecipitated fractions were probed with anti-cullins (CUL2 and CUL4B) antibodies.

We next examined whether Cif interacted with the neddylated forms of cullins. After infection of HeLa cells with EPEC expressing His-tagged Cif protein from *E. coli* (wild-type, Δ70-85 or C/A mutants), His-Cif proteins were immunoprecipited with anti-His antibodies coupled to magnetic beads and the different fractions were analyzed by western-blot (Fig. 6A). High molecular weight proteins detected with the anti-NEDD8 antibody co-eluted with the wild-type Cif protein. These data raised the possibility that Cif interacts with NEDD8-conjugated proteins. We next verified whether these neddylated proteins targeted by Cif were members of the cullin family. Figure 6B shows that many cullins (CUL1 to 4B) were found in the eluted fraction from the wild-type Cif precipitate. Interestingly, only the slowly-migrating neddylated forms of the cullins were recovered in the wild-type Cif eluates. These data suggest that Cif interacts with cullins through their NEDD8 modification. In contrast to our previous result showing that the Cif cysteine mutant interacted with NEDD8 *in vitro* (Fig. 1A–B), the Cif mutant did not co-elute with neddylated cullins in HeLa cells (Fig. 6B). However, after loading more concentrated amounts of the histidine-eluted fractions, western blot assays showed a weak (compared to wild-type His-Cif) but specific (compared to empty vector) signal using anti-cullin 2 and 4B antibodies in the His-Cif cysteine mutant immunoprecipitated fraction (Fig. 6C). Most likely, this weak signal reflects the low level of neddylated cullins observed in absence of active Cif, although it is also possible that the cysteine mutation partially impairs Cif ability to interact with neddylated cullins. These findings demonstrate that Cif induces the accumulation of neddylated cullins and also interacts with the NEDD8-conjugated form of these cullins.

Since cullins nucleate CRLs by recruiting a RING protein and substrate recognition module [27], we investigated which cullin-associated proteins are also pulled out of lysates by Cif. Analysis of immunoprecipited fractions showed that the RING protein subunit, RBX1, co-eluted with Cif (Fig. 6B). However, neither substrate adaptators for the CUL1-associated CRL, Skp1 and Skp2, nor the CRL inhibitor CAND1 (cullin-associated and neddylation-dissociated 1) were recovered in these fractions (Fig. 6B). Lack of CAND1 association is consistent with the fact that CAND1 only binds non-neddylated cullins [27], which are absent in the Cif eluate. The data also indicate that while Cif is able to interact with cullin-RING complexes via neddylated cullin subunits, these complexes may not contain substrate adaptor modules.

### Cif inhibits in vitro ubiquitylation activity of a NEDD8-modified CRL

**Figure 7:**
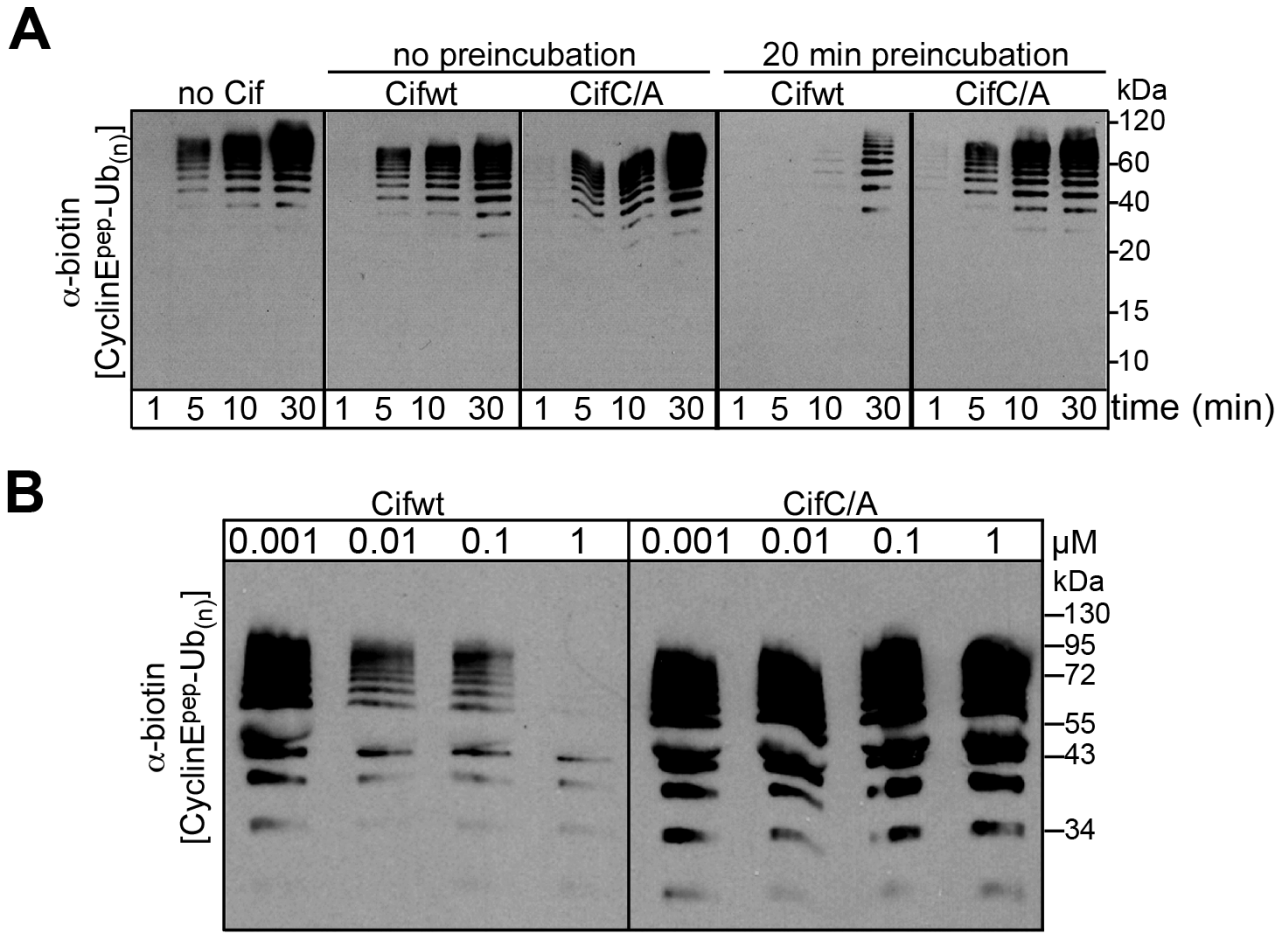
Cif decreases neddylated SCF-mediated substrate polyubiquitylation *in vitro*. (**A**) Anti-biotin western blot showing time courses of polyubiquitylation of a biotin-labeled cyclin E phosphopeptide by NEDD8-modified SCF^Fbw7ΔD^ in the presence or absence of purified His-tagged wild type Cif (Cifwt) or Cif catalytic Cys-to-Ala (CifC/A) mutant. Reactions were performed by adding Cif straight to the reaction (no preincubation), or after 20 min room temperature incubation of NEDD8-modified SCF^Fbw7(263-C)^ with Cif prior to initiating the reaction by adding all other proteins for polyubiquitylation activity (20 min preincubation). (**B**) Anti-biotin western blot showing titration of the indicated amount of Cifwt or CifC/A with 200 nM SCF^Fbw7ΔD^, prior to 10-min long polyubiquitylation reactions for a biotin-labeled cyclin E phosphopeptide. In all these reactions, the indicated version of Cif was preincubated with NEDD8-modified SCF^Fbw7ΔD^ for 1 h prior to reaction initiation.

Given that Cif associates with NEDD8-modified cullin-RING complexes, we wished to test the effects of Cif in an *in vitro* assay monitoring ubiquitylation activity of a neddylated CRL. We examined ubiquitylation of a biotin-conjugated phosphopeptide corresponding to a well-characterized degron from Cyclin E [28], [29], [30], [31]. When Cif is preinubated with a neddylated SCF^Fbw7^ (NEDD8∼CUL1-Rbx1-Skp1-Fbw7^ΔD^), ubiquitylation of the cyclin E phosphopeptide was inhibited in a manner that depended on the putative Cif active site cysteine (Fig. 7A). This inhibition also required preincubation of Cif with the neddylated CRL complex, prior to initiating the ubiquitylation reaction (Fig. 7A), indicating that Cif probably acts through a progressive process rather than an immediate effect. A titration experiment showed a concentration-dependent inhibition of *in vitro* ubiquitylation activity, with some inhibition observed even when the concentration of wild-type Cif was lower than that of the neddylated CRL (Fig. 7B). Thus, it seems likely that inhibition relies on Cif enzymatic activity toward the neddylated CRL, and not toward other components of the ubiquitylation reaction. Together, these data indicate that Cif acts directly to inhibit the neddylated CUL1-associated ubiquitin ligase activity.

### Cif blocks the degradation of various CRL substrates in cells

**Figure 8:**
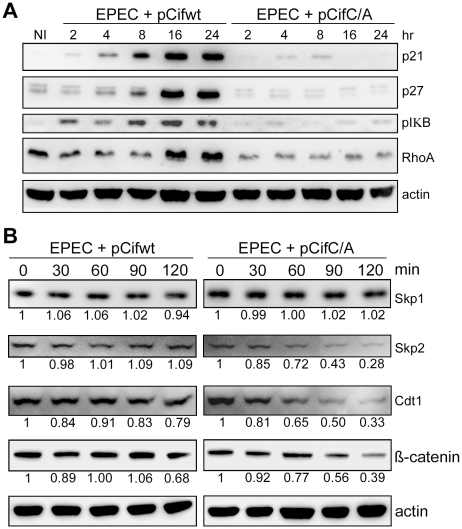
Cif induces stabilization of various targets of cullin-RING ubiquitin ligases. (**A**) HeLa cells were infected (starting at 0 h) for 2 h with EPEC*Δcif* carrying a plasmid encoding wild-type (WT) or cysteine mutant (C/A) Cif and further incubated for indicated times with antibiotics. Cell protein extracts were probed with anti-p21, p27, phospho-IκB, RhoA or actin antibodies. NI: non-infected. (**B**) HeLa cells were infected for 2 h with EPECΔ*cif* carrying the plasmid expressing cysteine mutant (pCifC/A) or wild-type Cif (pCifwt) and then (starting at 0 min) incubated for the indicated time with cycloheximide. Cell protein extracts were probed with anti-Skp1, Skp2, Cdt1, β-catenin or actin antibodies. The relative amount of protein (relative to 0 min and normalized to actin level) is indicated below each western blot.

Given Cif association with cullin-RING complexes, and inhibition of *in vitro* ubiquitylation activity for a CUL1-based CRL, we wondered whether Cif might exert such inhibition *in vivo* to block the degradation of various CRL substrates. To address this, the total levels of several known CRL substrates were monitored during a time course following HeLa cell infection with EPEC expressing the wild-type Cif or the Cif cysteine mutant. Western blot analyses showed that, p21, p27 (CUL1-Skp2 substrates), phospho-IκB (a CUL1-βTrCP substrate) and RhoA (a CUL3-BACURD substrate) accumulated in the presence of Cif (Fig. 8A). The total levels of other potential targets (Cdt1 and β-catenin) were only slightly modified in presence of Cif (data not shown). To evaluate the effect of Cif on these CRL substrates in a more sensitive assay, the half-lives of Cdt1 (a CUL4-DDB1-CDT2 substrate) and β-catenin (a CUL1-βTrCP substrate) were analyzed in the presence of the protein synthesis inhibitor cycloheximide. Both Cdt1 and β-catenin appeared to be stabilized by wild-type Cif, but not the cysteine mutant (Fig. 8B). We also examined the half-lives of substrate adaptors of CUL1, Skp1 and Skp2. In contrast to Skp1, whose level remained stable irrespective of the Cif status, the half-life of Skp2 increased in the presence of wild-type Cif (Fig. 8B). Altogether, these results demonstrate that Cif is able to control the stability of several key host cell regulators by targeting their cullin-associated ubiquitin ligases.

## Discussion

Cif proteins from vertebrate and invertebrate bacterial pathogens induce accumulation of p21 and p27 resulting in cell cycle arrest [6], [15]. Here, we have shown that Cif binds to NEDD8 and neddylated cullins, inducing their accumulation early after infection. Cullins are scaffold proteins nucleating Cullin-RING E3 ubiquitin-Ligases (CRLs), and neddylation activates cullins to modulate numerous cell functions, including cell cycle progression by targeting p21 and p27 for ubiquitin-dependent degradation [22], [32]. In this study, we show that Cif can directly inhibit the ubiquitin ligase activity of a neddylated CUL1-associated complex, which correlates with the Cif-mediated accumulation of p21 and p27. Consistent with Cif's association with different neddylated cullins, we observed a stabilization of substrates from a range of CRLs. The mode of action of Cif represents a novel strategy for modulation of a broad range of host cell functions by bacterial pathogens.

### Cif inhibits the ubiquitin ligase function of CRLs

NEDD8 conjugation of cullins is a major form of CRL regulation. Neddylation activates CRL-mediated substrate ubiquitylation by inducing a drastic conformational change of the C-terminal part of the cullin that frees the RBX1 RING, and thereby imparts catalytic geometries to an associated E2 enzyme [19], [27], [33]. Here, we report that Cif associates with the neddylated form of CUL1 to 4B, and that pre-incubation of Cif with a neddylated SCF^Fbw7^ complex inhibits its ligase activity. This latter result suggests that Cif acts on one or several components present in this reconstituted ubiquitylation assay. Whether Cif uses an enzymatic activity to usurp the CRL functions, and/or poisons the complex by binding to neddylated cullins is still elusive. However, the fact that preincubation of wild-type Cif with the neddylated SCF-Fbw7 is necessary to inhibit the ubiquitin ligase activity, and that Cif mutated on the cysteine residue of the catalytic triad is not inhibitory, support a predominant role for the putative enzymatic activity of Cif, which is (based on structure data) a member of the superfamily of cysteine protease, acetyl transferases, deamidases or transglutaminases. Interestingly, co-immunoprecipitation assays revealed an absence of association of Cif-bound CUL1 with Skp1-Skp2 adaptators. This Cif-induced loss of substrate recognition subunits would further lead to CRL inhibition *in vivo*. However, how Cif could induce dissociation/prevent association of Skp1-Skp2 from cullins, or how this effect is related to CRL inhibition by Cif remains to be elucidated.

### Cif induces cellular accumulation of neddylated cullins

CRLs transit through different stages of assembly, sequestration, neddylation, and deneddylation. To be fully active, CRLs undergo an activation cycle in which cullins oscillate between neddylated and non-neddylated (potentially sequestered by CAND1) states [27], [33]. Based on our results, we propose a model in which Cif interferes with this cycle and thus locks CRLs in a neddylated but inactive state. Cif appears to block many steps in the CRL activation cycle. First, we observe that CAND1, Skp1 and Skp2 are not associated with Cif-associated CRLs. Cullin neddylation may correlate with lack of CAND1 binding. Second, the levels of CRL substrates, and substrate-binding adaptors, may also influence cullin neddylation. A regulatory negative feed-back loop via cullin de-neddylation was reported to modulate CRL activity once substrate level decreases under a critical threshold [34]. Furthermore, Bornstein and collaborators reported that supplementation of Skp1-Skp2 and substrate to extracts of HeLa cells synergistically increased the level of neddylated CUL1 [35]. We found Cif-inhibition of CRLs also to correlate with the accumulation of numerous substrates and increased half-life of Skp2, which might secondarily result in accumulation of neddylated cullins, even though Cif-associated neddylated cullins are depleted of Skp1-Skp2 and presumably also inactivated. Finally, it is also possible that Cif interferes with cullin deneddylation. Since Cif treatment also inactivates neddylated cullins, their accumulation may essentially shut down both CRL assembly cycles as well as CRL ubiquitylation activities.

### Cif stabilizes numerous targets of CRLs

CRLs represent the largest subfamily of E3s and therefore play regulatory roles in numerous and diverse cellular functions. Since Cif binds to various neddylated cullins, we anticipated that, in addition to p21 and p27, Cif could modulate the stability of substrates of multiple different CRLs. Indeed, Cif induces not only cell cycle arrest but also stress fibers formation in certain cell lines [4], [6], [11], [12], [14]. A recent study showed that dysfunction of the CUL3-BACURD complex results in abnormal actin stress fibers and distorted cell morphology, owing to impaired ubiquitylation and degradation of the small GTPase RhoA [36]. Our results show that Cif inhibition/interaction with neddylated CUL3 correlates with accumulation of RhoA, thus providing a likely explanation for Cif-dependent stress fiber formation. We also investigated the stability of Cdt1 whose level is controlled by CUL4-DDB1-Cdt2 ligase complex [37], [38], [39]. Cdt1 is a licensing factor that plays a major role in the control of the S-phase [40]. We showed that Cif increases the half-life of Cdt1, thus providing a mechanistic link for the DNA re-replication that occurs in a fraction of HeLa cells 2 or 3 days post-infection [4], [14]. Cif also increases the stability of β-catenin whose level is important in the Wnt signaling pathway that is involved in homeostatic self-renewal in a number of adult tissues including intestinal epithelium, and in colon carcinogenesis [41]. Finally, we have also shown in this study that Cif inhibits the degradation of phospho-IκB, an inhibitor of the NF-κB pro-inflammatory response. Further experiments will be necessary to investigate thoroughly the role of Cif in these pathways and to evaluate whether this effector could participate to escape the host immune response and plays an important role in bacterial pathogenesis.

### Cif, a unique tool to study CRL regulation

Many bacterial toxins or microbial products mimic or modulate the activity of E3 ubiquitin ligases [42], [43], [44], [45]. For instance, deneddylation of CRL and consequently down-regulation of the NF-κB pathway have been proposed to explain the ability of intestinal bacterial communities to regulate the inflammatory tolerance of the mammalian intestinal epithelia [46], [47]. Similarly, a deneddylase encoded by Epstein-Barr virus promotes viral DNA replication by regulating the activity of cullin-RING ligases [48]. However, to our knowledge, inhibition of CRLs through a pathogen's protein binding to NEDD8 has never been reported in the pathogenesis of bacterial or viral infections. The requirement that CRLs be activated by NEDD8 conjugation on the cullin proteins offers an “achilles heel” for modulating this entire subfamily. Owing to this remarkable property, Cif appears as a unique tool to dissect the regulation of CRL-dependent ubiquitylation and neddylation processes and more generally, to investigate many host-signaling pathways including cell cycle regulation, DNA repair, immune and inflammatory responses, antigen processing or cytoskeleton dynamics.

## Materials and Methods

### Cell lines and bacterial strains

*Homo Sapiens* HeLa (ATCC CCL-2), and HEK 293T (ATCC CRL-11268) cell lines were cultured in Dulbecco's modified Eagle medium (DMEM; Invitrogen) supplemented with 10% fetal bovine serum (FBS; Eurobio) and 50 µg mL^−1^ gentamicin at 37°C in a 5% CO_2_ atmosphere. *Rattus Norvegicus* IEC-6 (CRL-1592) cells were grown in the above medium supplemented with bovine insulin (0.1 units mL^−1^; Sigma). *H. sapiens* HCT116 (ATCC CCL-247) were maintained in McCoy's 5A Medium (Invitrogen) supplemented with FBS and gentamicin as above. Bacterial strains and plasmids used in this study are listed in Table 1. Bacteria were cultured in lysogeny broth (LB) or in interaction medium (DMEM with 25 mM Hepes and 5% FBS). Antibiotics were used at the following concentrations: carbenicillin 50 µg mL^−1^, streptomycin 60 µg mL^−1^, chloramphenicol 20 µg mL^−1^ and kanamycin 25 µg mL^−1^.

**Table 1:** *E. coli* strains and plasmids used in this study.

Strains and plasmids	Genotype or description	Reference
**Strains**		
E22 wild-type	Prototype rabbit EPEC of serotype O103:H2	[52]
E22Δ*cif*	Rabbit EPEC Δ*cif::frt*	[4]
BL21 (DE3)	Laboratory *E. coli*	Stratagene
**Plasmids**		
pCDFDuet-1	Vector designed for coexpression of two target proteins	Novagen
pDuet- NEDD8	pCDFDuet-1 expressing NEDD8 protein	This study
pDuet- NEDD8 -Cifwt	pCDFDuet-1 expressing NEDD8 and His-Cif_Ec_ wild-type proteins	This study
pDuet- NEDD8 -Cif C109S	pCDFDuet-1 expressing NEDD8 and His-Cif_Ec_ C109S proteins	This study
pDuet- NEDD8 -Cif Δ70-85	pCDFDuet-1 expressing NEDD8 and His-Cif_Ec_ Δ70-85 proteins	This study
pDuet- NEDD8 -Cif C109S/Δ70-85	pCDFDuet-1 expressing NEDD8 and His-Cif_Ec_ C109S/Δ70-85 proteins	This study
pDuet- ubiquitin -Cifwt	pCDFDuet-1 expressing ubiquitin and His-Cif_Ec_ wild-type proteins	This study
pDuet- ubiquitin -Cif C109S	pCDFDuet-1 expressing ubiquitin and His-Cif_Ec_ C109S proteins	This study
pKTEM	TEM-1 fusion cloning vector derived from pBBR1MCS-2	[6]
pCifwt-TEM (pGJ626)	pKTEM expressing Cif_Ec_ -TEM fusion	[6]
pCifC109A-TEM	pKTEM expressing Cif C109A -TEM fusion	[10]
pCifΔ70-85-TEM	pKTEM expressing CifΔ70-85 -TEM fusion	This study
pGEX-4T-3	Vector designed to express GST-tagged recombinant proteins	GE Healtcare
pGEX-Cif C109S	pGEX-4T-3 expressing GST-Cif C109S fusion protein	This study
pFLAG-CMV- NEDD8	pFLAG-CMV vector expressing NEDD8	This study
pTRE-Tight	Vector for gene expression in Tet-On Tet-Off expression system	Clontech
pBRSK	Cloning vector derived from pBR328	[53]
pCifwt	pBRSK vector expressing Cif_Ec_ wild-type protein	[4]
pCifC/A	pBRSK vector expressing Cif_Ec_ C109A protein	[15]
pCifΔ70-85	pBRSK vector expressing Cif_Ec_Δ70-85 protein	This study
pCif_Bp_ (pEL1)	pBRSK vector expressing Cif_Bp_ wild-type protein	[6]
pET-Cifwt	pET28a vector expressing His-Cif_Ec_wild-type	[14]
pHB6 vector	Vector for HA N-terminal and His6 C-terminal fusion	Roche
pCifwtH6	pHB6 vector expressing a HA-Cif_Ec_-His fusion protein	[4]
pCifC/AH6	pHB6 vector expressing a HA-Cif_Ec_C109A-His fusion protein	This study
pCifΔ70-85H6	pHB6 vector expressing a HA-Cif_Ec_Δ70-85-His fusion protein	This study
pCif_Bp_wtH6	pHB6 vector expressing a HA-Cif_Bp_-His fusion protein	This study
pCif_Bp_C/AH6	pHB6 vector expressing a HA-Cif_Bp_C90A-His fusion protein	This study

### Generation of stable cell lines expressing GFP-Cif and GFP-Cif C109A

Fusion genes encoding GFP-Cif wild-type and C109A were amplified by PCR from plasmids pRN1 and pRN2 respectively [6] with primers pTRE-GFPCif-F (CGC CAC CAT GAG CGG GGG CGA) and pTagGFP-Cif-R (CGG GAT CCC TAA CTA CAT AGT GAT TTT ATT ATC TC). PCR fragments were digested with BamHI and ligated into the SmaI and BamHI sites of pTRE-Tight vector (Clontech). Both constructs were verified by DNA sequencing. After PvuI digestion, each plasmid was co-transfected with linear hygromycin selection marker into HeLa Tet-On Advanced cells (Clontech) using FuGENE6 transfection reagent (Roche) and cells were plated with the addition of 200 µg mL^−1^ of hygromycin B. Positive clones were identified by screening expression of GFP-Cif fusion proteins in presence of 2 µg mL^−1^ doxycycline inducer by fluorescence microscopy and western blot using anti-Cif and anti-Tag(CGY)FP antibodies (Evrogen).

### Yeast two hybrid screenings

Yeast two hybrid (Y2H) screenings were performed and analyzed by Hybrigenics (Paris, France). Briefly, wild-type Cif and Cif C109S mutant were PCR amplified and cloned into a Y2H vector optimized by Hybrigenics, checked by sequencing, and used as bait for Y2H screenings of both a human colon and a placenta cDNA libraries. Candidate prey fragments were sequenced and compared to the GenBank database using BLASTN. A predicted biological score was used to assess the reliability of each interaction. This score takes into account (i) the redundancy and independency of prey fragments as well as the distributions of reading frames and stop codons in overlapping fragments, (ii) the interactions found in all the screens performed at Hybrigenics using the same library. This score has been shown to positively correlate with the biological significance of interactions [49].

### NEDD8 binding assays

For bacterial co-expression experiments, the gene encoding NEDD8 was cloned into the second multiple cloning site of pCDFDuet-1 vector (Novagen) and genes encoding Cif proteins (wild-type or the mutants C109S, Δ70-85, C109S/Δ70-85) were cloned into the first multiple cloning site allowing expression of Cif tagged with a N-terminal hexahistidine motif. *E. coli* BL21(DE3) harboring the different constructs was grown at 37°C to OD 600 nm of 0.8, then induced with 1 mM IPTG and grown for an additional 3 h. The bacteria were lysed by sonication in presence of protease inhibitors (Complete, Roche). After centrifugation, bacterial extracts (supernatant) were incubated with Ni-NTA beads at 4°C for 16 h. Beads were washed four times with phosphate buffered saline (PBS) containing 0.1% NP40 and 20 mM imidazole. Bound proteins were eluted by resuspending beads with 60 µl of 3× SDS loading buffer and heating samples 10 min at 95°C for western blot analysis or with PBS buffer containing 250 mM imidazole for gel filtration chromatography analysis.

For GST pull-down, Cif C109S was cloned into the pGEX4T3 vector and the fusion protein GST-CifC109S was expressed in BL21(DE3) cells and purified using glutathione sepharose (GE Healthcare). HEK293T cells were transfected with pFLAG-CMV-NEDD8 using GenePORTER2 Transfection Reagent (Genlantis) following the manufacturer's protocol. 48 h post transfection, HEK 293T were lysed in RIPA buffer (50 mM Tris pH 8, 250 mM NaCl, 1% NP-40, 0.5% DOC, 0.1% SDS) in 3 freeze thaw cycles. Firstly, 100 µg of purified GST-CifC109S was bound to pre-equilibrated Glutathione Sepharose 4B in equilibrating buffer containing 20 mM Hepes pH 7.5, 5 mM MgCl_2_, 100 mM NaCl, and 2 mM DTT at 4°C for 1 h on a rotator. Either 100 µg or 250 µg of HEK 293T lysate was added to the GST-C109S bounded glutathione beads and further incubated at 4°C for 2 h on a rotator. The glutathione beads were washed once with equilibrating buffer, twice with wash buffer (20 mM Hepes pH 7.5, 300 mM NaCl, 5 mM MgCl2, 0.1% NP-40, 2 mM DTT), and lastly once with equilibrating buffer. 40 µl of 3xSDS loading buffer were added to the protein bound beads, and the beads were boiled at 95°C for 5 minutes. The samples containing only the supernatant were separated by SDS-PAGE and analyzed by immunoblotting membranes with anti-FLAG monoclonal antibody (Sigma).

### Infection and cell treatments

Infection experiments were performed as previously described [6]. Briefly, cells were infected 2 h with bacteria grown in interaction medium, with a multiplicity of infection (moi) of 50 bacteria per cell. After the infection, cells were washed with Hank's balanced salt solution (HBSS; Invitrogen) and cultivated for the indicated times in DMEM medium supplemented with 10% FBS and 200 µg mL^−1^ gentamicin. When needed, cycloheximide was added to the medium at 50 µg mL^−1^. For synchronization in G_1_/S phase, HeLa cells were treated with 2 mM thymidine (Sigma) for 18 h, washed 3 times with HBSS, incubated in normal medium for 9 h and treated again with 2 mM thymidine for 16 h.

### Actin stress fiber and cell cycle analyses

For cell morphology and actin cytoskeleton visualization, cells were fixed for 15 min in PBS 4% formaldehyde, permeabilized with 0.1% Triton X-100 and stained with rhodamine-phalloidin (Molecular Probes), DAPI (Sigma) or TO-PRO-3 (Invitrogen). Images were acquired with a DMRB fluorescence microscope equipped with a DFC300FX digital camera (Leica). For cell cycle distribution analyses, cells were trypsinized, washed, fixed with ethanol, stained with propidium iodide and analyzed using a FACScalibur flow cytometer (Becton Dickinson). For GFP quantification and cell cycle analyses of stable HeLa Tet On cells expressing GFP-Cif, cells were trypsinized, washed with ice-cold PBS, fixed for 3 h at 4°C in PBS with 1% formaldehyde, permeabilized overnight at 4°C in PBS ethanol 70%, stained with propidium iodide for 30 min at 37°C and analyzed by flow cytometry. Data from at least 20 000 cells were analyzed using FloJo software v8.5 (Tree Star).

### Cif and NEDD8 localization

Cells were grown and treated on LabTek glass slides, fixed with PBS 3.7% formaldehyde 15 min at room temperature, permeabilized with PBS 0.1% Triton-X-100 for 5 min, and blocked with PBS 0.1% Tween-20 1% bovine serum albumine (BSA) 1% normal goat serum. Slides were incubated overnight at 4°C with rabbit anti-NEDD8 antibodies (CST) or affinity purified anti-Cif antibodies [10] diluted 1∶200 in blocking buffer. Cells were washed and incubated 30 min at room temperature with affinity purified FITC or TRITC-conjugated goat anti-rabbit antibodies (Zymed) diluted 1∶150. After washing DNA was stained 5 min with TO-PRO-3 (Invitrogen) and the slides were mounted with Vectashield (Vector Laboratories). Images were captured with an Olympus-IX70 laser scanning confocal microscope using a ×60 PLANAPO NA = 1.4 objective. The confocal aperture was set for a z-axis thickness of 0.23 µm and images stacks were acquired in sequential mode, and analyzed with the Olympus FV500 FluoView software.

### Cell sample preparation and western blot analyses

For western blot analysis, 6×10^5^ cells were lysed in 80 µl of SDS-PAGE sample buffer, sonicated for 2 s to shear DNA and then boiled for 5 min. Cytoplasmic and nuclear extracts were obtained as described elsewhere [50]. For deneddylation assays, 50 µL of samples were treated with 0.5 µg of recombinant NEDP1 (NEDD8 protease 1; Biomol). Cell extracts were heated for 5 min at 100°C after addition of 4× Laemli loading buffer. Protein samples were resolved on 4–12% NuPage gradient gels (Invitrogen) and blotted on PVDF membranes. Membranes were blocked in TBST (10 mM Tris pH 7.8, 150 mM NaCl, 0.1% Tween20) 5% non-fat dry milk, then probed with primary antibody (0.5 µg mL^−1^) in TBST 5% non-fat dry milk. Primary antibodies were: anti-FLAG (Sigma), anti-NEDD8, anti-Cdt1, anti-lamin, anti-Skp1 (Cell Signaling Technology), anti-actin (ICN), affinity-purified anti-Cif [10], anti-p21, anti-p27, anti-Skp2, anti-14-3-3, anti-RhoA, anti-ubiquitin (Santa Cruz Biotechnology), anti-RBX1 ( = ROC1), anti-CAND1 ( = TIP120A) and anti-cullins 1 to 4B (Abcam). Bound antibodies were visualized with horseradish peroxidase-conjugated secondary antibodies. Acquisitions were performed with a Molecular Imager ChemiDoc XRS system (Bio-Rad).

### Immunoprecipitation assays

HeLa cells were infected with a moi of 100 bacteria per cell. After 3 h infection, cells were washed with HBSS and cultivated for 3 h in DMEM medium supplemented with 10% FBS and 200 µg mL^−1^ gentamicin and then were harvested in lysis buffer (50 mM Tris HCl pH 8, 150 mM NaCl, 1% Triton X-100, protease inhibitors). Extracts were processed according to manufacturer's protocol with µMACS anti-His microbeads (Miltenyi Biotec). Samples corresponding to total and non-retained (flow-through) proteins were collected during the immunoprecipitation protocol.

### Translocation assays

Translocation levels of Cif-TEM fusion proteins were determined using CCF2/AM (Invitrogen) as a substrate for intracellular TEM enzyme as described previously [6], [8]. Briefly, HeLa cells seeded in black 96-well plates were loaded for 1 h at 37°C with 1.7 mM of CCF2/AM diluted in DMEM with 2 mM probenecid and then infected for 2 h with bacteria expressing TEM fusion proteins. Fluorescence was quantified in a microplate reader (TECAN Infinite M200) with excitation set at 410 nm (9 nm bandwidth) and emission at 450 nm for blue fluorescence and 520 nm for green fluorescence (20 nm bandwidth). Translocation was expressed as the emission ratio at 450/520 nm.

### In vitro assays for neddylated SCF^Fbw7^-mediated polyubiquitylation

NEDD8 modification of SCF was carried out as previously described [19]. UBA1 was expressed as described [51]. GST-Fbw7^ΔD^ (i.e., the well-behaved monomeric Fbw7 containing residue 263 to the C-terminus) and Skp1 were coexpressed, and GST-Cdc34B, His-tagged Cif wild-type and Cif C/A were expressed in BL21 (DE3) Gold cells (Stratagene). GST-tagged proteins were purified by glutathione affinity, TEV or thrombin-cleaved and further purified by anion exchange and gel filtration chromatography. His-tagged Cif was purified by nickel-NTA affinity (Qiagen) and anion exchange chromatography. All proteins were desalted into 30 mM Tris-HCL, 20 mM NaCl pH 7.6. The neddylated SCF^Fbw7ΔD^ ubiquitylation substrate peptide was synthesized and purified by reversed-phase HPLC by the Hartwell Center for Bioinformatics and Biotechnology at St. Jude (Memphis, TN, USA), and corresponds to the high-affinity phosphodegron sequence from Cyclin E, with the sequence KAMLSEQNRASPLPSGLLT*PPQS*GRRASY (* = phosphorylation) [30], [31]. The peptide N-terminal sequence is acetylated, and the C-terminus is linked to a biotin moiety. Polyubiquitylation without Cif preincubation was assayed at room temperature with 200 nM NEDD8 modified SCF^Fbw7ΔD^, 0.5 µM Cdc34B, 50 µM ubiquitin, 1 nM–1 µM Cif as indicated (wild-type or catalytically-inactive Cys-to-Ala mutant), 2 mg mL^−1^ BSA and 5 µM biotin-conjugated cyclin E phosphopeptide in 30 mM Tris-HCL, 20 mM NaCl, 10 mM MgCl_2_, 5 mM ATP, pH 7.6, and started with 250 nM UBA1. Reactions were stopped with SDS sample buffer, and products were separated by SDS-PAGE, and visualized by western blot with anti-biotin antibodies (Rockland, 100-4198). For the preincubation time-courses, 1 µM Cif was mixed with 200 nM NEDD8 modified SCF^Fbw7ΔD^ for 20 min at room temperature prior to the addition of other reaction components, and reactions were stopped at the indicated times. For the titration experiments, a range of concentrations of Cif were incubated with 200 nM NEDD8 modified SCF^Fbw7ΔD^ for 1 h at room temperature prior to the addition of other reaction components, and reactions were stopped after 10 min.
